# The use of polyisobutylene-based polymers in ophthalmology

**DOI:** 10.1016/j.bioactmat.2021.09.005

**Published:** 2021-09-21

**Authors:** Leonard Pinchuk

**Affiliations:** a Distinguished Research Professor of Biomedical Engineering, University of Miami, Biomedical Engineering Dept., 13704 SW 92nd Court, Miami, 33176, FL, United States; b Founder and Senior Vice President, InnFocus, Inc., a Santen company, Miami, FL, United States

**Keywords:** Poly(styrene-*block*-isobutylene-*block*-styrene), SIBS, Glaucoma, MicroShunt, Intraocular Lens, IOL, Polyisobutylene, Cataract

## Abstract

A novel polyolefin called poly(styrene-*block*-isobutylene-*block*-styrene) (“SIBS”) originated from Joseph P. Kennedy's laboratory at the University of Akron (Akron, Ohio, United States) and was developed as a biomaterial for long-term implant applications by the author. SIBS has no cleavable groups on its backbone or sidechains, is comprised predominantly of alternating secondary and quaternary carbons on its backbone, which prevents embrittlement and cracking under flexion, and undergoes multiple purification steps which renders it extremely biocompatible and well-suited for long-term applications in the eye. This article explores two ophthalmic devices; 1) the PRESERFLO® MicroShunt (Santen Pharmaceutical Co. Ltd., Osaka, Japan) made from SIBS that lowers intraocular pressure to thwart progression of vision loss from glaucoma, and 2) a novel intraocular lens (IOL) made from crosslinked polyisobutylene, which is under-development by Xi'an Eyedeal Medical Technology Co., Ltd. (Xi'an, China) that does not glisten nor cloud over time, as do most conventional IOLs.

## Highlights

• A novel class of ultra-biostable polyisobutylene-based biomaterials for long-term implant applications.
• A novel class of polyisobutylene-based biomaterials that is not bioactive and elicits minimal foreign body reaction.
• A device called the PRESERFLO® MicroShunt to treat glaucoma made from poly(styrene-block-isobutylene-block-styrene) (SIBS).
• A novel crosslinked polyisobutylene material for intraocular lens applications that eliminates glistenings and halos.

## Abbreviations

4-VBCB: 4-benzylcyclobutene
*AGIS*: Advanced Glaucoma Intervention Study
*CE*: Conformité Européenne
*FDA*: US Food and Drug Administration
*HDCE*: hindered dicumylether
*IB*: isobutylene
*IDE*: Investigational Device Exception
*IOL*: intraocular lens
*IOP*: intraocular pressure
*MTF*: modulation transfer function
*OBC*: Optical Biophysics Center
*PDMS*: polydimethylsiloxane
*PIB*: polyisobutylene
*PMA*: premarket approval
*PMN*: polymorphonuclear leukocytes
*RI*: refractive index
*SIBS*: poly(styrene-*block*-isobutylene-*block*-styrene)
*Sn1*: unimolecular nucleophilic substitution
*xPIB*: crosslinked polyisobutylene
*xSIBS*: crosslinked poly(styrene-*block*-isobutylene-*block*-styrene)

## 1 Introduction

There are two polyisobutylene-based polymers discussed in this article. The first is poly(styrene-*block*-isobutylene-*block*-styrene) (“SIBS”), the material comprising a novel glaucoma drainage device. The second is crosslinked polyisobutylene (“xPIB”), a novel biomaterial that resolves many of the problems associated with current intraocular lenses (IOLs) used following cataract surgery.

Work on SIBS for long-term medical implants began in the early 1990s when Dr. Pinchuk's team at Corvita Corporation (acquired in 1998 by Pfizer (New York, New York, USA)) was attempting to develop a micro-porous synthetic vascular graft [1] made from a polyether urethane that was routinely used as electrical insulation on pacemaker leads [2]. The author observed that when these polyurethanes were spun into a micro-porous embodiment and implanted in animals, they persistently attracted granulocytes (*e.g*., macrophages, polymorphonuclear leukocytes (PMNs), and foreign body giant cells) as a consequence of their unintended biodegradation in the body. A review of this tissue reaction is published [3].

**Fig. 1:**
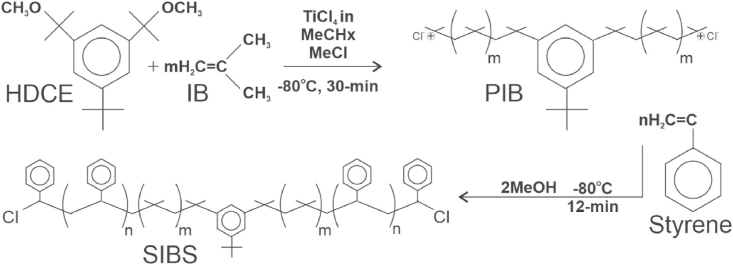
Synthesis of SIBS from an HDCE initiator. HDCE, hindered dicumylether; IB, isobutylene; PIB, polyisobutylene; SIBS, poly(styrene-block-isobutylene-block-styrene), MeCHx, methylene cyclohexane; MeCl, methylene chloride; MeOH, methanol.

The scientists at Corvita then set out to develop a biostable polymer devoid of degradable linkages (i.e., no urethane, ether, ester, carbonate, carbamate, amide, etc.) on either the backbone or side groups of the polymer. Fortunately, a family of such materials had been developed a decade earlier by Dr. Joseph P. Kennedy and his team at the University of Akron (Akron, Ohio, USA) [4]. The synthesis of SIBS is shown in Fig. 1. Corvita immediately licensed this family of polymers for implant applications and sought additional patents to protect new discoveries and applications [5,6]. Corvita then developed equipment and processes for stepping up and purifying the reactions for implantable applications.

The key feature in the University of Akron material is polyisobutylene “PIB”

–(CH_2_–C(CH_3_)_2_-)_n_-

which is a gum. PIB does not contain any labile linkages, and better still, as there is a dimethyl group on every second carbon (the quaternary carbon), the alternating secondary and quaternary carbon backbone cannot be oxidized to double bonds—the bane of many polyolefins such as polyethylene and polypropylene. The presence of double bonds on the backbone of polymers, especially when they are conjugated, leads to embrittlement, low flex fatigue life, and degradation.

In order to process the PIB gum into moldable or extrudable elastomeric medical devices, meltable glassy crosslinks are carbo-cationically polymerized onto both ends of the +PIB+ central block, in the form of polystyrene, to reversibly bind the amorphous (elastic/rubbery) PIB segments together [7]. The triblock polymer “SIBS” is shown in Fig. 1, where m is an integer greater than n. Variation in the molar ratio of PIB to polystyrene (m/n) changes the mechanical properties of the resultant triblock polymer; the more styrene (n), the more rigid the polymer.

**Fig. 2:**
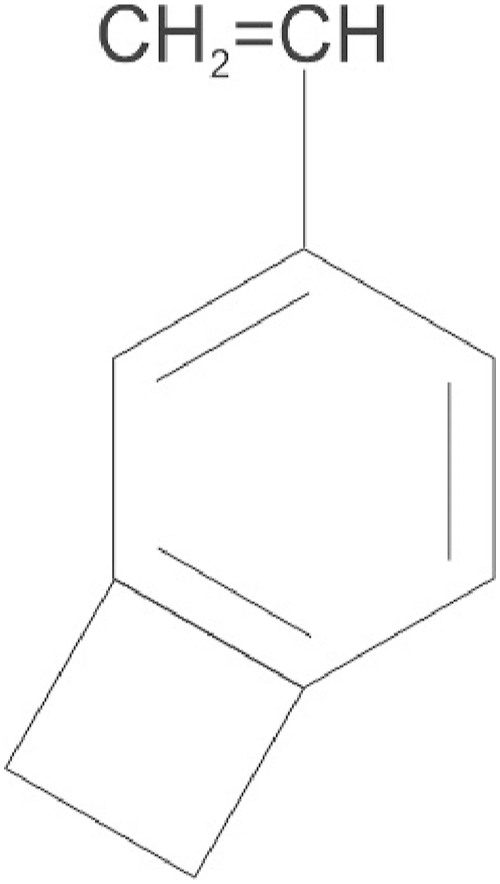
4-VBCB used as a crosslinker to thermally crosslink PIB or SIBS. 4-VBCB, 4-benzylcyclobutene; PIB, polyisobutylene.

**Fig. 3:**
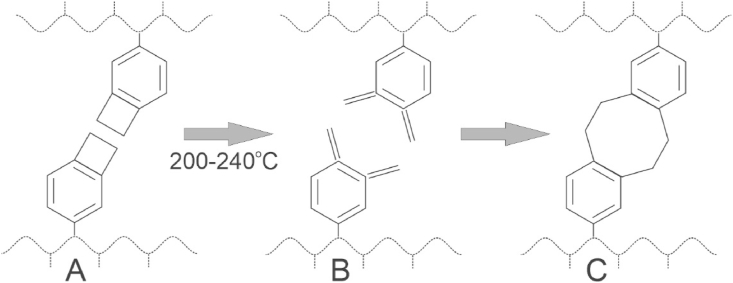
With heat, the stressed cyclobutene ring on the benzyl group in (A) opens to form a double bond intermediate (B) which reacts with another chain to form the crosslink (C).

Two deficiencies of the thermoformable SIBS triblock polymer that limit its use in certain embodiments are creep resistance, under both static and dynamic loading, as well as lipid absorption. In order to improve these properties, during the living-end synthesis of PIB or SIBS, the thermo-crosslinker 4-benzylcyclobutene (4-VBCB) (Fig. 2) is reacted into the growing chain to, upon the application of heat (200^o^-240 °C), yield the crosslinked polymer (xPIB or xSIBS) shown in Fig. 3 [8].

Once polymerized to a molecular weight of 40–60 KDaltons, and prior to crosslinking, the linear polymer is highly purified and vacuum-dried to a very thick sticky liquid/gum. It is then placed into the desired mold, and as shown chemically in Fig. 3, when the mold/polymer is heated, the stressed cyclobutene ring on the benzyl group in (A) opens up to form a double bond intermediate (B) that reacts with another similar chain in a ring-closure manner to form the 8-member crosslink shown in (C) [8]. Note that there is only one monomer, isobutylene, and that there is no byproduct from this crosslinking reaction. Nor are there residuals of initiator and the like to extract, which can affect the long-term clarity of a lens, as will be discussed below. The resultant polymer is ultraclean, three-dimensionally crosslinked, and biostable.

The first use of SIBS in medicine was for Boston Scientific Corporation's (Natick, Massachusetts, USA) TAXUS® Drug-Eluting Coronary Stent. The development of TAXUS® is well-documented in the literature [[9], [10], [11], [12]] and will not be repeated here.

## 2 The development of a glaucoma treatment device made from SIBS

**Fig. 4:**
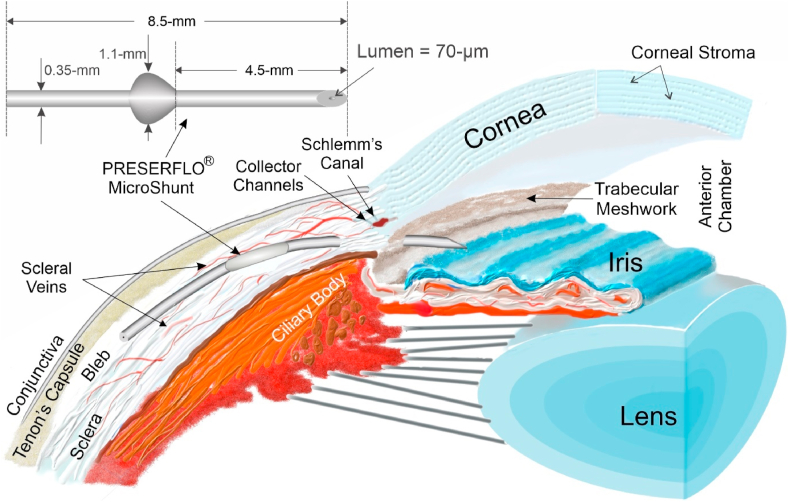
The PRESERFLO MicroShunt showing dimensions (top left) and placement in the eye shunting fluid from the anterior chamber to a bleb (small blister) formed between the conjunctiva/Tenon's capsule and the sclera.

Glaucoma is the second leading cause of blindness worldwide, with approximately 80 million people blind in 2020 due to this debilitating disease [13]. Glaucoma is a term describing a group of ocular disorders with multi-factorial etiology, united by a clinically characteristic intraocular pressure-associated degeneration of the optic nerve leading to blindness [14]. Data from AGIS (Advanced Glaucoma Intervention Study) suggested that intraocular pressure (“IOP”) must be reduced to the mid to low teens (<14 mmHg) to stop the progression of vision loss [15]. Referring to Fig. 4 for anatomy purposes, aqueous humor (the clear fluid in the front of the eye) is made in the ciliary body and is pumped between the iris and the lens through the pupil and into the anterior chamber. From there it drains through the trabecular meshwork into Schlemm's canal, then to the collector channels, then to the veins in the sclera (white of the eye), and then to the episcleral veins, to the retinal vein, and out the eye to the orbit and head. Any flow restriction in this pathway can lead to elevated IOP and glaucoma.

The most common method to reduce IOP—outside of medication and laser treatments—is to shunt aqueous humor from the anterior chamber to beyond the area where the outflow is restricted. Trabeculectomy has been regarded as the gold standard in glaucoma surgery since it was described in the 1960s to bypass these flow restrictions [16]. It is a surgical procedure requiring cutting a trapdoor in the sclera, punching a hole within this trapdoor to the anterior chamber, and removing a cylinder of sclera and some of the iris to form a channel (a fistula) connecting the anterior chamber to a natural dissection plane under the conjunctiva and Tenon's capsule. Aqueous humor flows into this space at a flowrate and pressure controlled by the tension in the sutures that keep the door of the trapdoor closed. However, while trabeculectomy is one of the most effective IOP lowering treatments, it requires much skill to perform and to set the proper suture tension as too much flow can lead to deflation of the eye and too little flow can elevate the pressure even further. Severe adverse events can occur, recovery can be prolonged, and intense post-operative management is required.

**Fig. 5:**
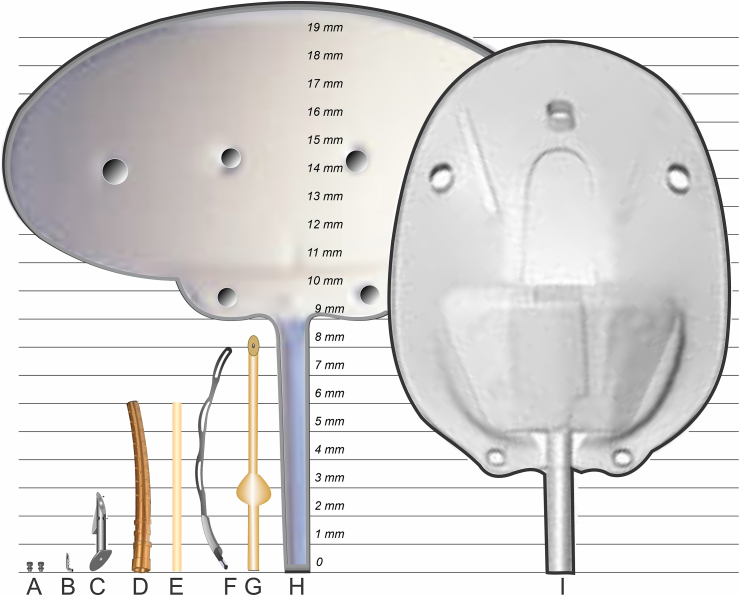
Various commercialized MIGS Devices: A) iStent Inject, B) iStent, C) Ex-Press Mini Shunt, D) Cypass Suprachoroidal Shunt, E) XEN Gel Stent, F) Hydrus Micro-Stent, G) PRESERFLO® MicroShunt, H) Baerveldt Glaucoma Drainage Implant, and G) Ahmed Glaucoma Drainage Implant.

In the early 2000s, entrepreneurs began to create medical devices in an attempt to obsolete trabeculectomy, which spurred a new “field” in the glaucoma arena called Minimally Invasive Glaucoma Surgery (“MIGS”) [17]. A comprehensive review of MIGS procedures was recently published in a book edited by Sng and Barton, and chapters in this book describe in detail each of the MIGS devices mentioned herein [18]. The devices, shown in Fig. 5(A) to (G), are MIGS devices and differ in size, function, and placement. Each device has its own benefits and deficiencies.

The iStent Inject (A) and iStent (B), both made from titanium (both from Glaukos Corporation, San Clemente, California, USA), and the Hydrus Microstent, made from nitinol (Ivantis, Inc., Irvine, California, USA), bypass high resistances in the trabecular meshwork. They are usually used in mild to moderate glaucoma patients and in conjunction with cataract surgery.. The Cypass Suprachoroidal Shunt (D) (Alcon Laboratories, Fort Worth, Texas, USA) is made from polysulfone and also bypasses the trabecular meshwork and directs aqueous humor to a secondary pathway draining from the eye. It was voluntarily recalled by Alcon due to occasional instances of touching the cornea, provoking endothelial cell loss that can lead to corneal opacification.

The remaining devices in Fig. 5 are generally used for more advanced glaucoma patients and, like trabeculectomy, bypass the trabecular meshwork, Schlemm's canal, the collector channels, and the veins in the sclera. These devices drain to a natural dissection plane under the conjunctiva and Tenon's capsule. When this plane fills with aqueous humor, it forms a blister-like bubble called a “bleb.” Fluid from the bleb drains to the episcleral veins or through the conjunctiva via microcysts or lymphatics to the tear film - whichever is the path of least resistance.

The Ex-Press shunt (C) (Alcon Laboratories, Fort Worth, Texas, USA) is made from stainless steel and is placed under the trapdoor of a trabeculectomy. It requires trabeculectomy-like surgery and control of IOP by lysing the sutures holding the door of the trapdoor closed. The XEN Gel Stent (E) (Allergan plc, Dublin, Ireland) is made from glutaraldehyde crosslinked porcine gelatin and is prone to scar formation, migration, and biodegradation [19,20]. However, when XEN functions correctly, it does provide an efficient means of controlling IOP [18].

The Baerveldt Glaucoma Drainage Implant (H), and Ahmed Glaucoma Drainage Implant (I) are made from silicone rubber or polypropylene and are relatively large devices with plates that are wedged between Tenon's capsule and the sclera (they are placed in the bleb). They are generally used in end-stage glaucoma. These devices (H and I) are not considered MIGS devices as they defy the term “minimally invasive.” They tend to reduce IOP significantly but are considered difficult and lengthy to implant and at times result in thick tissue capsules that limit eye motion, which can lead to double vision (diplopia) [21].

While all the aforementioned MIGS and large plate devices provide some reduction in IOP and relief from glaucoma medications, there is a need for a small plate-less glaucoma drainage device made from a polymer that will not degrade nor promote clinically significant scar tissue or encapsulation. As will be discussed in more detail below, the PRESERFLO® MicroShunt (G in Fig. 5), a SIBS-based glaucoma drainage device, satisfies many of these requirements. [The PRESERFLO® MicroShunt originated at InnFocus, Inc. (Miami, Florida, USA) in 2004 and was originally named the InnFocus MicroShunt. When InnFocus was acquired in 2016 by Santen Pharmaceuticals (Osaka, Japan), the name of the product was changed to the PRESERFLO MicroShunt.]

The development of a SIBS-based glaucoma treatment device to stop the progression of glaucoma was a joint effort between InnFocus, Inc. and the University of Miami's Miller School of Medicine, Bascom Palmer Eye Institute, Optical Biophysics Center (OBC, Miami, Florida, USA) [22]. The OBC team implanted 1 mm thick SIBS disks in the corneal stroma, as well as under the conjunctiva and Tenon's capsule, of New Zealand white rabbits (see Fig. 4 for anatomy). Control disks made from silicone rubber (polydimethylsiloxane “PDMS”) punched from the plate of a Baerveldt Glaucoma Drainage Implant (Fig. 5 (H)) were implanted alongside the SIBS disks. The results of the two-month implants were published by Acosta et al*.* [23], who showed that there were no myofibroblasts or angiogenesis in the vicinity of the SIBS disks, nor were there integral capsules surrounding the disks. In contrast, angiogenesis, myofibroblasts, and significant capsules surrounded the silicone control disks. The authors concluded that SIBS was found to be very inert and innocuous in the eye.

Subsequent conversations with glaucoma experts led to the hypothesis that if a tube shunting aqueous humor from the anterior chamber to under the conjunctiva and Tenon's capsule (i.e., to a bleb) did not encapsulate, then it might not require a massive plate. It could be used as a treatment to lower the pressure in the eye and thwart glaucoma-induced vision loss. To shunt aqueous humor from the anterior chamber to under the conjunctiva, the tube needed to be at least 8 mm long. The lumen serves as a flow restrictor to prevent the pressure in the eye from dropping to below approximately 6 mmHg for a prolonged period, as this deflation of the eye (hypotony) could lead to choroidal or retinal detachment and other serious sequelae. The lumen diameter was approximated from the Hagen Poiseuille equation [24] to be approximately 70 μm, and a series of rabbit eye implants by Arrieta et al. and Fantes et al. [25,26] confirmed that a nominal lumen diameter of 70 μm did indeed satisfy these requirements. For columnar strength to facilitate pushing the device into the eye through a needle tract, the outer diameter of the tube was set at 350 μm.

A fin was added to the tube halfway down its length, as a simple tube without a fin often migrated through the needle tract into the anterior chamber. Another benefit of the fin is to serve as a “cork” to prevent fluid leakage around the tube rather than through the lumen by sealing the needle tract, which can be larger than the tube. Fig. 4 shows a schematic of the PRESERFLO® MicroShunt and its placement in the eye. It took approximately five years to optimize (2006–2011) the design and placement of the tube, the surgical procedure, and the drug regimens used before, during, and after implantation. The clinical course is reviewed in detail by Pinchuk et al. [27] and in the book by Sng and Barton [18]. A recent comparison of the MicroShunt with trabeculectomy in the rabbit model was published by Fujimoto et al. [28].

Preclinical bench testing of the MicroShunt was intensive, as would be expected for a long-term implant and is presented in detail in the literature [9,22]. Briefly, sterilized MicroShunt samples were incubated in distilled water at 37 °C for 14 days. Dimensional changes due to swelling were slight (between 1.0% and 4.5%) and fell within specified tolerances. Hydrolysis accelerated testing was performed using MicroShunt facsimiles that were incubated in distilled water at either 55 °C for 15 months, 85 °C for 57 days, or 100 °C for 20 days, for the equivalent of five years of real-time exposure. The aged samples showed no changes in appearance and no measurable weight change (to 0.00001 g precision), indicating no evidence of hydrolytic instability. Three-year aged samples, subjected to Soxhlet extraction with isopropyl alcohol for 4 h, showed <0.2% weight change. Analysis of the eluent showed only trace levels of low molecular weight siloxanes, ethylene glycol, benzophenone, 2-phenylphenol, and low molecular weight alkyl polyol contaminants; virtually all of these contaminates originated from the Mylar/Tyvek packaging materials and were considered insignificant. Biocompatibility studies were completed in accordance with ISO 10993-1-2009 recommendations. All biocompatibility testing suggested that the devices were sufficiently safe to proceed to human trials.

Professor Isabelle Riss, of Pôle Ophtalmologique de la Clinique Mutualiste, Pessac, France, was the first surgeon to implant a first prototype of the MicroShunt in humans in January 2006. A review of her work and a description of four independent clinical trials that led to the final design and method of implantation is presented by Pinchuk and Riss et al. [22,27,29].

A 23-patient feasibility trial with 0.4 mg/mL mitomycin C as an intraoperative antifibrotic, applied for 3 min, was initiated in the Dominican Republic by Dr. Juan Batlle in patients with primary open angle glaucoma and no previous conjunctival incisions, who had failed maximum tolerated glaucoma medication [30]. Implantation into patients with no previous surgery, using a dose of 0.4 mg/mL Mitomycin C, led to a qualified success rate of 100%, with a 55% drop in intraocular pressure (IOP) from baseline at one year [30]. A success rate of this nature with a synthetic tube was previously unheard of in the glaucoma community. A 2021 publication by Batlle et al. confirms that the MicroShunt is still performing well in the majority of patients at five years [31]. Early data from this latter study by Batlle et al. [30] helped support the granting of a Conformité Européenne (CE) Mark on January 9, 2012, enabling the device to be commercialized in Europe, as well as a U.S. Investigational Device Exception (IDE) by the U.S. Food & Drug Administration (FDA), which was granted in May 2013 with enrollment completed in 2019 [32]. At the time of this writing, over 17,000 PRESERFLO® MicroShunts have been implanted in humans. The device is approved for sale in Europe, Australia, New Zealand, and Canada; FDA approval for sale in the United States is pending at the time of this writing.

In summary, the development of the PRESERFLO® MicroShunt was an educated, iterative process that occurred over the course of a decade. The development process required sophisticated chemistry and engineering, including controlling the foreign body reaction with SIBS. Draining to under the conjunctiva and Tenon's capsule in the posterior part of the eye, as opposed to the anterior Tenon's capsule adjacent to the limbus where trabeculectomy is performed, is relatively novel in glaucoma surgery. The bleb formed more posteriorly is thicker-walled and potentially less prone to adverse events, compared with the thin-walled blebs formed in the anterior part of the eye where trabeculectomy is performed. On the other hand, Tenon's capsule is thicker in the posterior part of the eye [33] with more fibroblasts and smooth muscle cells requiring a higher dose of an antiproliferation medication, such as mitomycin C, to optimize outcomes [34,35]. In several studies, the MicroShunt has lowered IOP by 30–55% from preoperative baseline to below 14 mmHg and reduced the need for glaucoma medications, with no long-term sight-threatening adverse events [18,30,31,[36], [37], [38]]. According to the Advanced Glaucoma Intervention Study (AGIS), the control of IOP to a level below 14 mmHg suggests that glaucomatous progression of vision loss will be unlikely [15].

## 3 A novel crosslinked polyisobutylene (xPIB)-Based intraocular lens

Opacification of the natural lens in the eye (called a cataract) is the leading cause of blindness worldwide [55]. Following surgical removal of the cataractous lens, an intraocular lens (IOL), which is usually a rigid or semi-rigid lens, is placed in the lens capsule of the eye to restore vision. Sir Harold Ridley was the first to successfully implant an intraocular lens (IOL) in the human eye on November 29, 1949 [39].

**Fig. 6:**
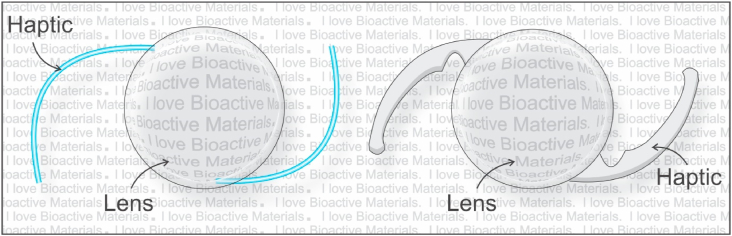
Schematic of two generations of IOL: Left historic 3-piece IOL with whisker haptics. Right, foldable single piece IOL. IOL, Intraocular lens.

Fig. 6 is an illustration of an historic three-piece IOL (left) and a state-of-the-art single piece IOL (right). The whiskers, or arms, on either side of the lens are called “haptics” and serve to keep the lens centered in the lens capsule. The evolution of the three-piece IOL to the single piece IOL was driven by several factors as follows: The three-piece lens requires an “assembly” of the three pieces which is more labor intensive and therefore more expensive to manufacture. Conversely, the single-piece lens can be injection-molded or reaction-injection-molded in a single mold or machined from a “button” of the appropriate material, or combinations of the above; that is, the optic can be molded, and the haptics machined from a skirt around and integral to the optic. The single piece design also opened the door to different shaped haptics that helped keep the lens both centralized and parallel to the iris in the lens capsule.

These first rigid IOLs, such as those made by Sir Harold Ridley were made from polymethylmethacrylate (PMMA) [39]. Silicone rubber (PDMS) and acrylic foldable lenses became accepted by the cataract surgery community around 1998 [40]. (As elucidated below, the term “acrylic” is actually a misnomer; however, I will continue to use this term for consistency with the literature.) A foldable lens can be folded in half along its long axis and loaded into and deployed from a cannula. A non-foldable 6 mm diameter lens requires at least a 6 mm incision into the eye to place it, whereas a foldable lens reduces this incision size to under 3 mm, requiring less to no suturing. However, there are still some unmet needs for these devices that must be addressed: (1) glistening and hazing; (2) the observance of halos at night; (3) the need to implant through a small incision; and (4) compatibility with silicone oils – where certain lenses, such as those made from silicone elastomer, can absorb silicone oils from vitrectomy (removal of the vitreous humor and replacement with silicone oil) [[41], [42], [43]].

A novel IOL made entirely from crosslinked polyisobutylene (xPIB) that cannot hydrolyze or oxidize *in vivo* originated in an InnFocus, Inc. sister company named Innolene LLC (Miami, Florida, USA). The Chinese patent for this formulation was sold to a Chinese entity in 2014 which later became known as Xi'an Pillar Bioscience Co., Ltd. (Xi'an, China). Pillar management trademarked the name Eyedeal® IOL for the lens and changed the name of the company in 2021 to Xi'an Eyedeal Medical Technology Co., Ltd. (Xi'an, China) (”Eyedeal”). The chemistry of xPIB is shown in Fig. 3. For IOL applications, it is necessary to bond an ultraviolet (UV) blocker into the polymer to keep UV light from damaging the retina.

At the time of this writing, prototype xPIB IOLs have been tested in rabbit eyes alongside controls of a state-of-the-art acrylic lens (AcrySof IOLs from Alcon, Fort Worth, Texas, USA). As expected, the tissue reaction surrounding the xPIB lens was found to be benign and minimal and indistinguishable from the controls (results not published). A formal rabbit study confirming these findings is currently in progress and publications are expected once the study is complete. However, an early assessment of the findings related to the xPIB lens by Professor Gerd Auffarth suggests that the Eyedeal IOL may satisfy many of the unmet needs of currently commercialized lenses [41].

### 3.1 Glistening and hazing

It is well recognized that hydrophobic acrylic IOLs, which are the most implanted IOLs, glisten and haze with time [44,45]. Glistening is a formation of star-like specs or bright spots that occur in the lens. Hazing, also at times called “clouding” or “whitening,” is a slow progressive opacification of the lens that is usually unnoticeable to the recipient, but it does affect contrast sensitivity at night. Although ophthalmologists and regulatory agents are highly aware of glistening and hazing, and they do not like to see it, there are few, if any, commercially available better alternatives. Colin et al. report the prevalence of glistening or whitening to be as high as 60% in examined eyes [45]. Miyata et al. show that this phenomenon is observable, on average, 6.6 months post implantation [46]. Hazing in explanted IOLs (AcrySof MA60BM [Alcon, Texas, USA]) implanted for five to 11 years manifests as a mild opacification that originates slightly below the surface of the IOL, and glistening bright spots are present near the center of the lens [44]. The investigators also report that when these lenses are dried out, they become clear; however, when immersed in physiological saline, whitening recurs within one to 3 h [44]. This quick reappearance of glistening/whitening strongly suggests that chemical changes have occurred in the lens that are sites for absorbed water.

**Fig. 7:**
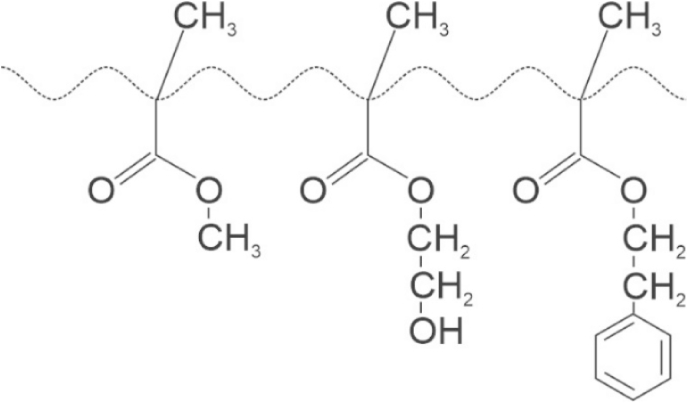
Acrylic IOLs (technically methacrylate IOLs) often comprise combinations of (from left to right) methylmethacrylate, 2-hydroxyethylmethacrylate, and 2-phenylethylmethacrylate IOL, intraocular lens.

A simplified chemical structure of the acrylic lens polymer is shown in Fig. 7. Due to the presence of ester groups on their sidechains, these “acrylic” lenses are technically “acrylates.” Further, due to the “methyl” group on the quaternary carbon on the backbone *and* the ester group on the sidechain, they are more appropriately “methacrylates.” However, to be consistent with the literature, I will continue to use the term “acrylic” to describe this family of “methacrylate” IOLs. The most common monomers (Fig. 7) are methylmethacrylate, 2-hydroxyethylmethacrylate, and 2-phenylethylmethacrylate [41]. The ratios of these comonomers differ among manufacturers and exact formulations are proprietary. It is noteworthy that formulations that contain more 2-hydroxyethylmethacrylate tend to be more hydrophilic due to the presence of the hydroxyl group on the sidechain.

Acrylates, by nature of their intrinsic ester groups on their sidechains, can be cleaved by hydrolysis even at body pH and temperature in the absence of base by the slow unimolecular nucleophilic substitution (Sn1) reactions that occur over time. These hydrolysis reactions are probable because the byproducts (e.g., methanol, ethylene glycol, and 2-phenylethanol, respectively) are excellent leaving groups, which are favored in Sn1 reactions. Upon cleavage, the resultant moiety on the acrylate polymer becomes a carboxylic acid that can imbibe water, and the differences in the index of refraction (1.33 for water and 1.54–1.55 for acrylics) turn the material cloudy and eventually opaque. As the acrylic lenses are generally hydrophobic, water does not readily enter the interior of the lens. However, as Matsushima observed, the whitening manifests on the surface of the IOL [44]. As discussed below, it requires only one-to-two hydrophilic groups per mole of polymer to turn xPIB white and opaque. There is a plethora of ester groups per mole of acrylate polymer that can cleave.

Clues to the causes of clouding in acrylic IOLs came from an observation by this author that long-chained polyisobutylene:

–(CH_2_C(CH_3_)_2_)_n_-

of molecular weight >60,000 Da, which is all aliphatic and devoid of heteroatoms, does not cloud or glisten in aqueous media; however, when the ends of the polymer are hydroxylated to form the diol:

HO–(CH_2_C(CH_3_)_2_)_n_-OH

the polymer readily clouds in aqueous media. This unpublished experiment clearly demonstrated that as few as two polar groups per long-chain PIB are sufficient to cause whitening or clouding.

It is also hypothesized that when hydrophilic groups are formed in the acrylic materials, the polymer can reorient with the hydrophilic groups, grouping together to form micelles which, in the presence of interstitial water, pool sufficient water to form vacuoles that can manifest as glistening or clouding. These vacuoles can increase or decrease in size with osmotic pressure. These vacuoles may be what Saylor et al. described in their experiments [47]. When an empty micelle, or microdomain of methacrylic acid-containing polymer, is formed in the polymer, and the polymer matrix is exposed to water, water will migrate into the micelle, which will swell up and manifest as glistening. When the polymer matrix is exposed to a higher osmolar medium such as saline, there is no reason for pure water to want to leave the saline and migrate to the micelle, as water migrating away from saline will only concentrate the saline that it is leaving, which is contrary to the laws of thermodynamics. Therefore, the less solute in the surrounding media, the more swelling of the polymer. This observation is consistent to what Saylor et al. [47] described in their studies (i.e., the higher the osmolarity, the less swelling). In addition, polymers comprising methacrylic acid when dry are extremely brittle. This may account for the brittle skeletons also observed by Saylor et al. Still further, Saylor et al. looked thoroughly for impurities to be removed by the aqueous media, which were never found, again implicating bound acid or polar groups as the hydrophilic moiety and possible source of glistening and hazing.

Another factor that can lead to glistening in the acrylic IOLs is differential swelling which can occur if homopolymerization of any of the starting monomers occurs. For example, if polymethylmethacrylate homopolymer is formed during the reaction, it will swell less than the bulk polymer system, which contains a mixture of copolymers including the swellable hydrogel, poly(2-hydroxyethylmethacrylate). In this case, water will imbibe into the polymer in the annular space between polymethylmethacrylate and the bulk polymer, which may account for the sporadic glistenings observed when the hydrated IOL is cycled through modest temperature cycles. Other factors that contribute to glistening include trapped or extracted initiators, unreacted monomer, and oligomer, which can leave voids where water can accumulate when dissolved out of the crosslinked matrix. In addition, these impurities can also swell differently from the bulk if trapped in the matrix. The difference in index of refraction between the imbibed water and the bulk polymer would manifest as hazing or whitening.

In summary, evidence indicates that the acrylics/acylates/methacrylates which currently comprise the majority of implanted IOLs glisten and whiten with time. The current theory is that insoluble particles in the matrix swell differentially from the bulk matrix, which allows gaps or vacuoles to fill with water. In the longer term, disassociation of esters from the sidechains of these polymers results in methacrylic acid on the sidechain, which can imbibe water, cause hazing, eventually opacify the lens, and, when dried out, resemble brittle skeletons. In addition, this process will begin from the surface of the lens and migrate towards the bulk. On the other hand, ultra-pure xPIB-based polymers do not have dissociable moieties anywhere on the backbone or side groups and therefore do not show glistenings, whitening, or hazing.

### 3.2 Halos, crescents, and glare

Approximately 20% of patients who undergo cataract surgery suffer from positive (e.g., halos, glares, flashes, streaks of light) or negative (e.g., shadow in the visual periphery) dysphotopsias [48]. Halos, crescents, and glare are caused by the following interacting factors and stimuli: (1) diameter of the IOL relative to the diameter of the pupil; (2) sharp edge of the IOL; and (3) glistenings [[49], [50], [51]]. If the IOL has a smaller diameter than the pupil, unfocused light will travel around the periphery of the IOL and be perceived as a halo. Similarly, if the IOL is decentralized, unfocused light will shine on the retina in a crescent shape. The sharp edge of an IOL, as well as glistenings, can reflect or scatter light and be perceived as glare.

In optics and lens design, the Abbe number is a measure of the material's dispersion or aberration (variation of refractive index versus wavelength), with high Abbe numbers indicating low dispersion. High RI materials (1.52 is considered high) usually have low Abbe numbers, and high Abbe number materials (50 is considered high) usually have low RI. The xPIB polymer has an Abbe number of about 50 and a refractive index (RI) of approximately 1.52. As shown in Table 1, xPIB is unique in that it has a well-balanced, relatively high RI and a high Abbe number, which manifests as better light transmission (modulation transfer function [MTF]) than many other IOL materials. A possible rationalization by Dr. Zhou Yang, from Eyedeal, for why PIB has both a high RI and Abbe number is presented in the footnote below.^1^ Also contributing to its excellent light transmission and clarity of the xPIB IOL is the use of a single monomer (isobutylene) with only one refractive index as opposed to acrylic IOLs with multiple monomers with several different refractive indices that may interfere with each other.

**Table 1:** Refractive index (RI) and Abbe number of common hydrophobic IOLs.

IOL models	Pillar Bioscience (Xi'an, China) PX65AS1	Alcon (Fort Worth TX) AcrySof IQ SN60WF	PrecisionLens (Bloomington MN) Tecnis ZCB00	Bausch & Lomb (Rochester NY) EnVista MX60
Material	xPIB	Hydrophobic Acrylic	Hydrophobic Acrylic	Hydrophobic Acrylic
RI	1.52	1.56	1.47	1.54
Abbe	50	37	52	41

RI = refractive index, Abbe = Abbe number.

Table 2, compiled by Eyedeal scientists, presents the properties of the xPIB IOL compared with Alcon's SN60WF IOL. The higher the RI, the more the magnification, and the thinner the IOL needs to be for a specific diopter (magnification). In addition, the high elongation and low modulus of xPIB allows more compression (and elongation) in an inserter cannula and a smaller inserter required for a similar geometry IOL.

**Table 2:** Comparison of Pillar xPIB IOL to Alcon Acrylic SN60WF (Alcon, Texas, USA).

Characteristics	Pillar xPIB PX65AS1	ALCON SN60WF
Material	xPIB	Acrylic
Chemical bond	C, H	C, H, O
IOL glistening	Glistening free <12 Miyata scale	>60 Miyata scale
Refractive index	1.52	1.56
Material ABBE no.	50	37
White light MTF	>0.56	∼0.45
Material elongation	>300%	120–150%
Incision size (mm)	≤2.0	2.2–2.6
Optic body (mm)	≥6.5	≤6.0
Clear optic (mm)	6.5	6.0

*IOL,* intraocular lens; *MTF*, modulation transfer function; *xPIB*, crosslinked polyisobutylene.

### 3.3 Microincision versus larger lens

Traditional IOLs (e.g., acrylic) are typically 6.0 mm in diameter and are most often inserted into the lens capsule through a 2.3 mm diameter cannula. Experiments performed by Eyedeal engineers demonstrated that a thick 6 mm diameter, 35 diopter xPIB lens can be inserted into the lens capsule using a canula of only 1.8 mm diameter. These engineers faced a dilemma of whether to reduce the canula diameter to 1.8 mm, which would reduce the incision size and be arguably less traumatic to the patient, or to increase the xPIB IOL diameter to 6.5 mm to ensure better coverage of the pupil and reduce the incidence of halos and crescents, which would require the use of the larger conventional 2.3 mm canula. The engineers decided to proceed with the 6.5 mm diameter xPIB IOL using a conventional 2.3 mm canula, as halos and crescents were among the most common patient complaints post-operation. State-of-the-art commercialized acrylic lenses risk scratching if enlarged to 6.5 mm diameter and forced through a 2.3 mm inserter canula.

### 3.4 Compatibility with silicone oils

Silicone oils are often used following vitrectomy. Unfortunately, certain IOL materials can absorb silicone oil and become distorted or cloudy. By nature of its chemistry, xPIB is not known to be affected by silicone oil and therefore should not be contraindicated in patients who have undergone vitrectomy with silicone oil, or who are expected to undergo this type of vitrectomy [43].

In summary, the combination of an xPIB lens being larger than the pupil and blocking peripheral light, the balanced RI and Abbe number, the lack of glistening and hazing, and the design of the xPIB lens all contribute to providing an ideal IOL that can be introduced through a conventional inserter and provide exceptional clarity with few, if any, halos, crescents, glares, or other aberrations found in state-of-the-art acrylic IOLs. The non-degradable nature of xPIB and lack of leachable impurities should prevent glistening and hazing over time. Publications documenting these virtues are forthcoming.

## 4 Concluding remarks

Both *in vitro* and *in vivo* testing confirmed the biocompatibility and biostability of SIBS [10,11,23,30]. At the time of this writing, a drug-eluting coronary stent (TAXUS® Boston Scientific Corporation, Natick, Massachusetts, USA) and a glaucoma device (PRESERFLO® MicroShunt) are the only commercialized implantable medical devices made from SIBS [9]. TAXUS was the largest product launch in the history of medical devices with sales exceeding $3 billion USD in the first year alone. Since the year 2000, SIBS-coated paclitaxel-eluting coronary stents were implanted in over six million hearts (per data from discussions with Boston Scientific officers). SIBS was the cornerstone that led to the success of this product. The PRESERFLO® MicroShunt has been implanted in over 17,000 eyes since 2006, with the vast majority of implants occurring in Europe commencing in 2020. This number is expected to grow significantly upon FDA approval in the United States. Both the drug-eluting coronary stent and the PRESERFLO® MicroShunt have changed the practice of medicine for these two extremely debilitating diseases. It is expected that the Eyedeal IOL will be commercialized in China in 2022 or 2023 and the rest of the world soon thereafter.

At present, researchers developing medical devices utilizing polyisobutylene-based polymers (stents, glaucoma drainage devices, IOLs, and heart valves [9]) have found the following: (1) SIBS does not substantially activate platelets in the vascular system [9]; (2) PMNs in large numbers are not commonly observed around SIBS implants subcutaneously in the vascular system [12], in implants, or in the eye [23]; (3) myofibroblasts, scarring, and encapsulation are not clinically significant with SIBS implanted in the eye [[23], [24], [25], [26], [27]]; (4) embrittlement has not been observed in any implant location [27]; (5) insignificant calcification has been observed within this polymer during *in vitro* studies [52] with no reports of calcification of any device from the clinical field; and (6) degradation has not been observed nor reported in any living system to date. Areas where SIBS is deficient include areas in direct contact with fat, where the lipids can absorb into the polymer to plasticize and weaken it. Similarly, contact with ointments containing parabens should be avoided as they, too, soften the polymer, and SIBS exhibits creep deformation under high static and/or dynamic load. The crosslinked forms of xPIB and xSIBS are not as affected by lipid and paraben absorption, and their static and dynamic creep behavior are much improved [9]. Polyisobutylene-based polymers have been investigated in other areas of the eye including scleral buckles [53] and as orbital tissue expanders [54]. These applications were abandoned when analyses showed that the cost to convert these devices from silicone rubber to polyisobutylene-based and the associated cost of regulatory approval were higher than the potential market revenue. Lastly, Xi'an Eyedeal Medical Technology Co., Ltd. (Xi'an, China) is considering commercializing polyisobutylene-based polymers for world-wide distribution and may be contacted for further discussion.

## CRediT authorship contribution statement

**Leonard Pinchuk:** The author is an inventor of SIBS in medicine, the PRESERFLO® MicroShunt as well as the Eyedeal Lens. All three inventions are discussed in the manuscript. The author wrote the manuscript as well as several of the references.

## Declaration of competing interest

The author is an employee of Santen Pharmaceutical Co. Ltd., Osaka, Japan, which owns the glaucoma device described herein. The author is also the Chief Scientific Consultant for Xi'an Eyedeal Medical Technology Co., Ltd. (Xi'an, China), which is developing the intraocular lens described herein.

## Footnotes

1 It is well-known that the RI increases with aromatic and polar moieties. However, there are minuscule aromatic groups from the crosslinker in PIB and there are no polar groups. The weight percentage of the quaternary carbon in PIB is 21.4%, which is one of the highest of any organic polymer and the RI is 1.52 and Abbe number 50. Diamond, which is 100% quaternary carbon has an RI of 2.42 and an ABBE number of 55, which suggests that the more quaternary carbons as a percentage of the monomeric weight, the higher the RI as well as the ABBE number. In comparison, the acrylic IOLs contain quaternary carbons, polar groups and aromatic comonomers and therefore have high RI's of approximately 1.55 but the percent quaternary carbon per comonomer is lower, e.g., the comonomers methylmethacrylate is 12%, 2-hydroxyethylmethacrylate is 9.2% and 2-phenylethylmethacrylate is 6.2%, therefore the Abbe number of the combination would be less than PIB and is measured for the AcrySof IOL to be 37. Light is dispersed less when the percent quaternary carbon is higher which seems to correlate with Abbe number.
